# Reduction of surgical complications via 3D models during robotic assisted radical prostatectomy: review of current evidence and meta-analysis

**DOI:** 10.1007/s11701-024-02041-2

**Published:** 2024-08-06

**Authors:** Kenaan Sarhan, Nawal Khan, Davide Prezzi, Michela Antonelli, Eoin Hyde, Findlay MacAskill, Christopher Bunton, Nick Byrne, Andres Diaz-Pinto, Armando Stabile, Alberto Briganti, Giorgio Gandaglia, Nicholas Raison, Francesco Montorsi, Sebastien Ourselin, Prokar Dasgupta, Alejandro Granados

**Affiliations:** 1 School of Biomedical Engineering and Imaging Sciences, King’s College London, London, UK; 2 Medical Physics and Clinical Engineering, Guy’s and St Thomas’ NHS Foundation Trust, London, UK; 3 Department of Urology, Guy’s Hospital, London, UK; 4 Unit of Urology, San Raffaele Hospital, Milan, Italy; 5 Innersight Labs, London, UK; 6 NVIDIA, Santa Clara, CA USA

**Keywords:** Robot assisted radical prostatectomy, 3D-printed models, 3D-virtual models, Surgical complications

## Abstract

The use of 3-dimensional (3D) technology has become increasingly popular across different surgical specialities to improve surgical outcomes. 3D technology has the potential to be applied to robotic assisted radical prostatectomy to visualise the patient’s prostate anatomy to be used as a preoperative and peri operative surgical guide. This literature review aims to analyse all relevant pre-existing research on this topic. Following PRISMA guidelines, a search was carried out on PubMed, Medline, and Scopus. A total of seven studies were included in this literature review; two of which used printed-3D models and the remaining five using virtual augmented reality (AR) 3D models. Results displayed variation with select studies presenting that the use of 3D models enhances surgical outcomes and reduces complications whilst others displayed conflicting evidence. The use of 3D modelling within surgery has potential to improve various areas. This includes the potential surgical outcomes, including complication rates, due to improved planning and education.

**Supplementary Information:**

The online version contains supplementary material available at 10.1007/s11701-024-02041-2.

## Introduction

Prostate cancer is the second most common cancer amongst men globally [1]. A robot-assisted radical prostatectomy (RARP) is indicated for men with localised and non-localised disease and an acceptable life expectancy [2]. As expected with any surgical procedure, a RARP might result in complications, which studies have shown to find. An example is Novara et al., who conducted a systematic analysis of common complications following a RARP procedure [3]. Lymphocele/lymphorrhea were the highest reported (3.1%), with urine leak reported at 1.8% and reoperation at 1.6% [3].

Diagnosis and management of prostate cancer is increasingly supported by multi-parametric magnetic resonance imaging (mp-MRI) [5], and yet, the size and extend of prostate tumours are consistently underestimated on medical images [6]. Compared to traditional MRI sequences, mp-MRI is beneficial for a better identification of cancer lesions and typically consists of 3-dimensional (3D) T2-weighted imaging (T2w), diffusion-weighted imaging (DWI) with apparent diffusion coefficient (ADC) and dynamic contrast-enhanced (DCE) sequences.

However, despite the quality and resolution of MRI images that allow inspection of the anatomy, a comprehensive understanding of a patient’s true anatomy and pathology is often limited due to the 2-dimensional (2D) formatting [4]. A good understanding of the anatomy is essential preoperatively to ensure adequate surgical planning to reduce the chance of complications occurring perioperatively. Currently, surgeons are expected to mentally reconstruct a patient’s anatomy in 3D from the 2D slices composing an MRI image presented to them [4]. Although this mental reconstruction becomes less challenging with experience, the issue remains for juniors and in difficult cases. Surgical management of prostate cancer is determined by the anatomical location and its association with the neurovascular bundle and prostatic capsule. In this context, improvement of 3D appreciation of the tumour’s anatomy; including size, number and location of lesions—may be useful [7]. Therefore, this gap has led to the recent generation and use of 3D patient-specific models in surgery [4]. Tack et al. conducted a systematic review, using three literature databases to gather studies that investigated the impact of 3D-model intervention when used as anatomical and surgical guides [8]. Two hundred and twenty-seven papers were included in the final review, 86 of which found an improvement in clinical outcomes and 28 reporting reduced operative times [8].

This literature review will focus on the use of 3D-printed and/or -virtual models in preoperative planning and perioperative guidance and the effect they have in reducing surgical complications after a RARP. It aims to provide a comprehensive overview of the current research available on the use of 3D models in the RARP procedure, detailing their findings, the advantages, and disadvantages of the models—and any limitations to these trials conducted. These findings will allow us to conduct research effectively and contribute to the current knowledge in the field.

## Materials and methods

### Search strategy

PubMed, Medline and Scopus electronic databases were selected to gather relevant research. Key terms including “3D models”, “prostatectomy”, and “complications” were used as different domains to gather further topics (Table 1—Supplementary Material). Topics derived from each of these domains were then trialled as search terms across databases to gather the number of hits. The final search terms found to gather the most relevant number of papers were “*3D models*” and “*robotic assisted radical prostatectomy*” (Table 2—Supplementary Material). After the initial papers were gathered, they were screened for duplicates using an automated method through Microsoft Endnote X9, then manually double-checked for duplicates. Once duplicates were removed, each title was screened for relevance. If the title was not sufficient to determine the relevancy, then the abstract was manually screened and if further needed, the paper itself. The inclusion–exclusion criteria were then applied and those papers that did not match the criteria were removed (Table 1). Both randomised control trials and clinical trials were included in our search. This process was done independently by two reviewers.

**Table 1:** Inclusion–exclusion criteria used in the study and the justification for each criterion

Inclusion	Exclusion	Justification
Research published between January 2013 and May 2023	Research published before January 2013	This is a relatively new researched field with a limited number of research—therefore using a wider timeframe of 10 years allows for as much research as possible to be reviewed for relevance
Research published in English language	Research published in another language with no English translation available	No available translation into English
Studies conducted on humans	No human trials	This research question can only be answered by trials on human patients
Primary studies that are RCT/clinical trials	Anything that was not an RCT or clinical trial	Scientific design that allows for the impact of 3D models with the caveat of possible bias

### Post-operative outcomes

The following postoperative outcomes were investigated. Oncological outcomes were represented by positive surgical margins. Surgical outcomes were represented by complications. Functional outcomes were represented by continence recovery and erectile potency recovery, both at 6 and 12 months after surgery.

### Statistical analysis

We conducted a meta-analysis and created forest plots in R© software version 4.4.1 (The R Foundation for Statistical Computing) for each of the postoperative outcomes described above. We grouped studies according to their type and reported risk ratio (RR) and its confidence interval (CI), common and random effect models, overall prediction, and between-study variance/heterogeneity for binary outcomes (tau).

## Results

**Fig. 1:**
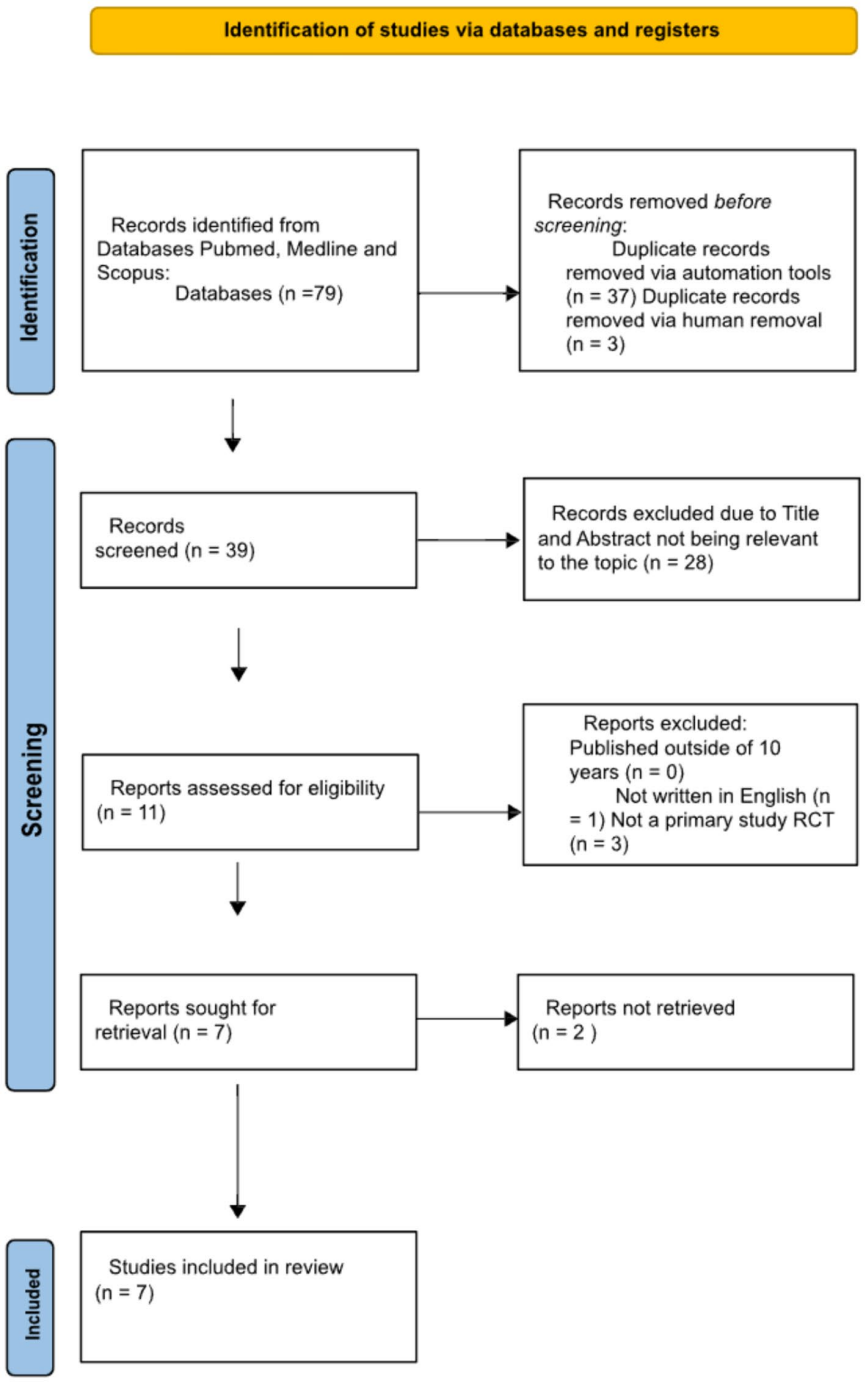
Prisma flow diagram presenting the results from the literature search

Systematic searches of the three included databases returned 79 studies. Following exclusions, seven studies were included in the final analysis (Fig. 1). Seven papers were gathered through this literature review to evaluate and determine the evidence related to the effect 3D-printed [7, 9–14] and 3D-virtual models [10–12] used during RARP have on the reduction of surgical complications. We summarise the results of our analysis on all the studies resulting from this literature review in Table 2. A total of 1075 patients participated in all seven studies.

**Table 2:** Summarising the studies obtained from the literature review

Study	Aim of study	Study type	Number of participants	Anatomy	Printed/virtual	Software	Positive resection margins	Urinary incontinence	Erectile dysfunction
Wake et al. [9]	Surgical outcomes (PSM%, blood loss, and operating time)	Cohort	*N* = 82 *C* = 47 *I* = 35	Prostate gland and tumour, rectal wall, urethra and bladder neck, and neurovascular bundles	Printed	Mimics 20.0 (image segmentation)	Positive effect on patient outcomes with 3D-model intervention PSM control = 22.2% PSM intervention = 8.1% *p* = 0.13	N/A	N/A
Oderda et al. 2022 [10]	Surgical strategy, outcomes (PSM%)	Cohort	*N* = 11	Not specified	Virtual	Koelis trinity (3D-US-mp-MRI elastic fusion)	Maximisation of functional outcomes can be achieved without increasing the risk of PSM with 3D-TRUS-mp-MRI elastic fusion imaging PSM = 0%	N/A	N/A
Shirk et al. [11]	Surgical strategy, surgical, functional outcomes (PSM%,, incontinence, potency, PSA)	RCT	*N* = 92 *C* = 51 *I* = 41	Prostate gland and tumour, bladder, urethra, neurovascular bundles, seminal vesicles, iliac arteries and iliac veins	Virtual	Ceevra reveal (image segmentation)	Improvement in oncological outcomes whilst maintaining functional outcomes with 3D model PSM control = 33% PSM intervention = 25% p = 0.4	6 months post-op Control = 1.4 pads/day Intervention = 0.9 pads/day *p* = 0.4	6 months post-op SHIM score Control = 11.7 Intervention = 11.1 *p* = 0.8
Checcucci et al. [12]	Surgical and functional outcomes (nerve sparing, catheterisation time, length of stay, PSM%, continence, potency)	Cohort	*N* = 800 *C* = 160 *I* = 640	Not specified	Virtual	Medics3D (image segmentation)	3D model favours the use of nerve sparing approach and aids in limiting PSM% PSM control = 35.1% PSM intervention = 25% *p* = 0.01	12 months post-op Control = 95.5% Intervention = 96.8% *p* = 0.2	6 months post-op Control = 36.2% Intervention = 38.7% *p* = 0.6
Bianchi et al. [13]	Surgical outcomes (index lesions, console time, blood loss, nerve sparing, and PSM’s)	Cohort	*N* = 40 *C* = 20 *I* = 20	Prostate gland, index lesion, urinary sphincter, and NVBs	Virtual	D2P software (image segmentation and virtual models)	3D AR models have potential to allow for a reduced PSM at the index lesion PSM control = 20% PSM intervention = 15% *p* = 0.2	12 months post-op Control = 95% Intervention = 95% *p* = 0.2	6 months post-op Control = 57.1% Intervention = 66.6% *p* = 0.06
Porpiglia et al. [14]	Surgical and functional outcomes (blood loss, console time, continence, nerve sparing, catheterisation time, length of stay and postoperative complications)	Cohort	*N* = 40 *C* = 20 *I* = 20	Not specified	Virtual	HA3D (Virtual model)	Elastic 3D AR models when correctly simulated can reduce PSM and increase functional outcomes PSM control = 35% PSM intervention = 25% *p* = 0.73	1 month post-op Control = 85% Intervention = 90% *p* = 1	N/A
Chandak et al. [7]	Surgical and functional outcomes (biochemical recurrence, continence, potency)	IDEAL phase 2a	*N* = 10	Prostate gland and tumour	Printed	Mimics medical v18 (image segmentation)	Promising development however models do not represent normal tissue elasticity and add extra costs PSM intervention = 10%	12 months Intervention = 100% continence	Of seven patients who had a pre-op SHIM OF > 21, four patients achieved strong erections at 24 months

*RCT* randomised control trial, *N* total number of participants, *C* number of participants in control group, *I* number of participants in intervention group

Blood loss and operating/console time were recorded in four studies with conflicting results [7, 10–12]. Across all four studies there were no significant differences in operating time or blood loss. Whilst Wake et al. and Checcucci et al. noted small reductions in operating time, Porpiglia et al. and Bianchi et al. reported longer surgery times. Wake et al. reported a 9-min reduction in operating time (213 ± 42 min vs. 222 ± 47 min) and 5 ml reduction of blood loss (227 ± 148 mL vs. 232 ± 114 mL) in the 3D-printed model group, differences that were not statistically significant (*p* > 0.5) [9]. Operating time was lower for the 3D-virtual group with a mean of 113 vs 118 (*p* = 0.07) in the study by Checcucci et al. [12]. In contrast, Bianchi et al. found that the augmented reality (AR) 3D-virtual intervention group did have a longer console time and higher blood loss, 216 min and 200.2 ml in comparison to 208 min and 182.5 ml in the non-intervention group (*p* > 0.06) [13]. Porpiglia et al. found similar results with an increased operating time in the 3D-virtual group—140 vs. 117.5 (*p* = 0.10) and a higher blood loss for the 3D group 240 vs. 230 ml (*p* = 0.55) [14].

Interestingly, a change in surgical strategy was reported by Oderda et al. and Shirk et al. [10, 11]. Oderda et al. found that 3D-virtual modelling with the Koelis trinity system was feasible and required a very limited time to be performed successfully, 6 min per patient median. Modelling changed surgical strategy in six cases where bilateral nerve sparing was performed as opposed to unilateral [10]. Shirk et al. found in 32% of cases, surgeons re planned their operation based on seeing the virtual model. In addition, the 3D-virtual group had increased nerve sparing in comparison to the control group (78 vs. 92%) [11]. Checcucci et al. described similar results with increased nerve sparing in the 3D-virtual group vs. control with a full nerve spare in 20.6 vs. 12.7%, intermediate in 38.1 vs. 38.0% and standard in 41.2 vs. 49.2% [12]. Porpiglia et al. did not report any cases of full nerve spare; increased nerve sparing was observed in the 3D-virtual group vs. the control group with a partial nerve spare in 85 vs. 35% and a minimal nerve spare in 15 vs. 65% [14]. Bianchi et al. did not note any significant difference in nerve sparing for both groups (*p* ≥ 0.3) [13].

Post-operative complication rates were reported by Checcucci et al. and found to be comparable (*p* = 0.9) for patients in the 3D-virtual and control groups with 5.6% (9 patients) vs. 4.8% (31 patients), respectively [12]. On the contrary, Bianchi et al. found more complications in the 3D-virtual AR group compared to the control group, i.e. two vs. one patient [13]. Porpiglia et al. documented complications according to Clavien–Dindo gradient under level 3 being lower for 3D-virtual group (one vs. two) and Clavien–Dindo complications over level 3 higher in the 3D-virtual group (one vs. zero), although results were not statistically significant [14]. Improved urinary continence was reported in the intervention group (1.4 pads vs. 0.9 pads per day) by Shirk et al. [11]. However, four studies including Shirk, Checcucci, Porpiglia and Bianchi et al. found no significant difference in functional outcomes [11–14]. Chandak et al. reported continence (no pads) in four/ten patients immediately after surgery, seven/ten at 3 months, eight/ten at 6 months, and all by the year in his 3D-printed model cohort. Potency scores were noted to be above 21 pre-op for seven patients of which four patients achieved strong erections strong enough for intercourse at 24 months [7]. Shirk et al. reported that when the 3D-virtual group was compared to the control group there was a lower post-op and a detectable PSA (31 vs. 9%, *p* = 0.036). Chandak et al. did not observe any biochemical recurrence.

In terms of positive surgical margins (PSM), all studies showed a nominal decrease in PSMs with the use of 3D models with only Checcucci et al. reporting a statistically significant improvement [9–14]. Wake et al. showed a PSM rate of 22.2% for the imaging group and 8.1% for the 3D-printed model group with a p value of 0.13 [9]. Shirk et al. reported lower PSM rates in the intervention group (33 vs. 25%) and Checcucci et al. 25% in 3D-virtual group vs. 35.1% in non 3D (*p* = 0.01) [11, 12]. Porpiglia et al. reported a similar result of 25% in the intervention group vs. 35% in the control group (*p* = 0.73) [14]. PSMs were found to be lower in the study with Bianchi et al. with the intervention group 15 vs. 20% and PSMs at the index lesion were significantly lower in the AR 3D group—5 vs. 20% [13]. Chandak et al. reported a PSM in one out of ten patients however, this was not compared to any control group and Oderda et al. did not have any PSMs to report [7, 10].

**Fig. 2:**
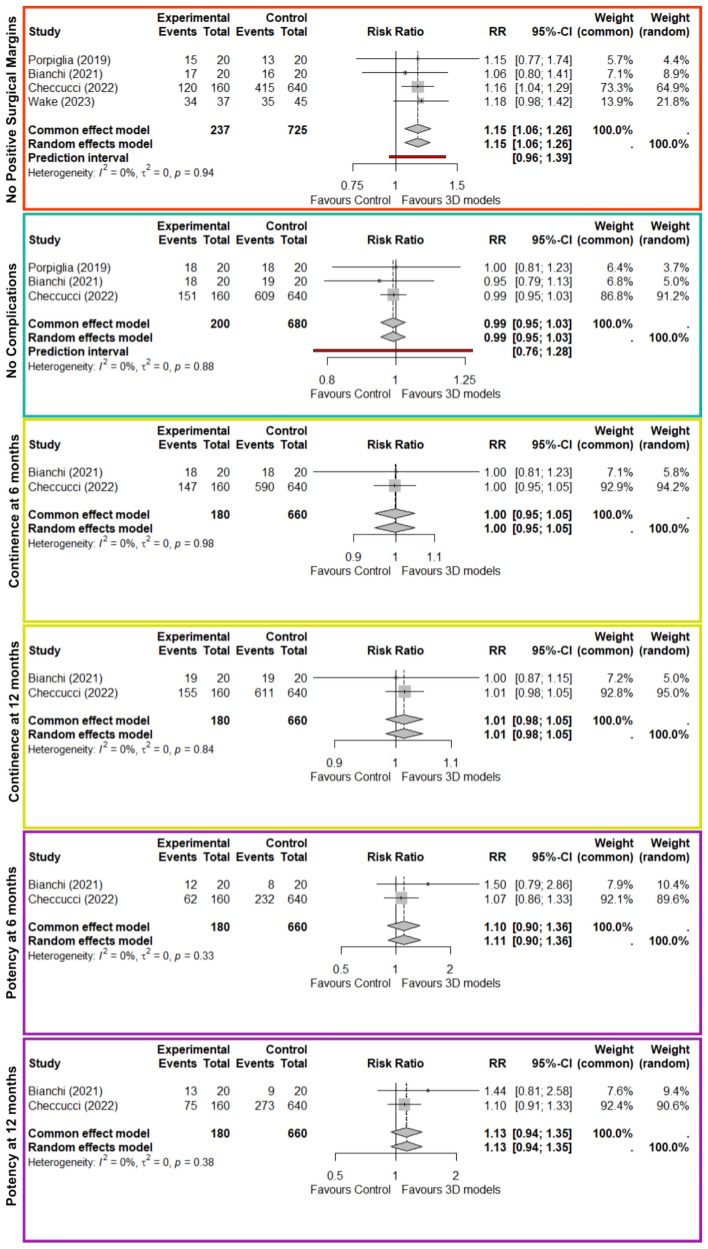
Meta-analysis of studies stratified by no positive surgical margins (row 1), no postoperative complications (row 2), urinary continence at 6 and 12 months (rows 3 and 4, respectively), and erectile potency at 6 and 12 months (rows 5 and 6, respectively)

Meta analysis did not demonstrate any significant difference between-study heterogeneity (${I}^{2}=0\%$, $p\ge 0.33$). Pooled analysis of surgical margins rates showed a significant benefit with 3D models ($\text{RR}=1.15;\text{CI}=[1.06-1.26]$) (see Fig. 2—row 1). However, 3D models did not show any statistically significant benefits for complications or functional outcomes at 6 or 12 months (see Fig. 2—rows 2–6). We observed confidence intervals for the summary estimate of fixed and random effects models are similar across outcomes.

## Discussion

Currently, surgeons are expected to mentally reconstruct a patient’s 3D anatomy from the 2D imaging presented to them [4]. This reconstruction becomes less challenging with experience, although it remains an issue for juniors and in difficult cases. This gap in surgery has led to the use of 3D patient-specific models in the form of either virtual or printed models [4]. Although virtual models have been developed to help enhance anatomical understanding, they still have drawbacks since they rely on dedicated software and individuals to be able to construct them Moreover, 3D-virtual models technically remain as flat images projected onto a screen, requiring the surgeon to rotate the images to visualise their 3D representation [4].

3D-printing technology has been proposed to overcome these challenges. The benefits of using 3D-printed models relate to the sensory and perceptual information exchange resulting from tactile feedback [4]. However, 3D-printed models can take longer than virtual models to manufacture due to having to produce the physical model.

Investigating the effect that 3D-printed/virtual patient-specific models have on the reduction of complications in the context of RARP is captured throughout this review. Research in this area is increasing and showing evidence that there is a positive impact on surgical margins and functional outcomes, despite the limited number of studies found in recent literature.

### 3D-printed models

Wake et al. assessed surgical metrics after the use of a 3D-prostate model. The models used were in fact printed—using mimics 2.0 software to segment them [9]. Wake et al.’s findings did overall suggest a trend that the models do improve surgical outcomes and decrease complications, although with limited statistical significance. PSM was significantly lower for the 3D model at 8.1% compared to 22.2%, a difference deemed statistically insignificant [9]. A 9-min reduction in operative time was seen for the intervention group—a surrogate metric implied to be correlated with lower chance of complications; differences were not statistically significant [9]. Moreover, the blood loss decreasing by 5 ml for the intervention group was also statistically insignificant [9]. Although these results do not entail the ineffectiveness of these models, more robust studies are necessary to understand the mechanisms leading to a reduction of complications, if any. Other studies, such as Porpiglia et al., reported findings that also support the lack of direct improvement based on using models [14]. However, overall, qualitative data from the surgeons did show to be positive and that surgeons were more confident based on the use of the 3D models [9].

Chandak et al. also used 3D-printed models for their phase 2a IDEAL study [7]. All RARPs were carried out by the same surgeon and the models were also produced via the same software of Mimics 2.0 [7]. Similar to Wake et al., Chandak et al. did find positive outcomes from the intervention of 3D models. All patients in this study were followed up for a year post-op and recovery was reported to be remarkable. Continence measured via the use of no pads was seen immediately in four patients and sequentially by seven in 3 months, 8 in 6 and all 10 by the year [7]. Four patients found to have reported to gain an erection strong enough for intercourse by the year [7]. All patients also found the use of personalised counselling useful [7]. This study, like that of Wake et al., did find positive results that support the use of 3D modelling in reducing surgical complications and improving outcomes—however the extent to whether this can be accredited to the models is limited.

### 3D-virtual models

Shirk et al. generated 3D-virtual models using Ceevra Reveal software [11]. These models consisted of the prostate, lesion mass, bladder, urethra, neurovascular bundles, seminal vesicles, iliac arteries and veins [11]. Models would then be viewed on a smartphone and Virtual Reality software and on the robotic control screen [11]. Shirk et al. found a decrease in PSM rates from 33 to 25% and reported better urinary continence in the intervention group—similarly to how Chandak et al. found better urinary continence recovery. There were no significant differences in PSA, SHIM and Nerve sparing scores—limiting the extent to which models potentially decrease complications. Surgeons replanned their procedure after seeing the model [11].

Checcucci et al. evaluated the impact of 3D-virtual models in PSM post-op and oncological outcomes [12]. They report lower complications in the 3D group (*p* = 0.9), which supports Wake et al. and Chandak et al. for using 3D models to decrease surgical complications [12]. Furthermore, similar findings did include a lower operative time for the 3D-model group, lower PSM group—regardless of the model type, i.e. cognitive or augmented [12]. However, on the contrary to the aforementioned studies, Checcucci et al. did not find any difference in continence and potency recovery between the two groups [12].

Porpiglia et al. aimed to look at their new 3D elastic augmented reality AR system and compare it to 2D standard imaging [14]. Contradictory to Checcucci et al., this study suggests there is no relationship between models and complications. This study found that blood loss was higher for the 3D group [14]. In addition, they also reported higher complications with a Clavien complications score above 3 in the 3D group (1 vs. 0) [14]. On the other hand, there were less complications in the 3D group with a Clavien score of below 3, 1 vs. 2, and there was also a decreased operative time in the 3D group [14].

Bianchi et al. conducted a prospective study investigating the use of an AR model [13]. This model consisted of the prostate gland, index lesion, neurovascular bundles, and urinary sphincter—significantly less structures in comparison to Shirk et al. [11, 13]. The 3D group was found to have an increased operative time, 216 min vs. 208 min, and an increase in blood loss, 200.2 ml vs. 182.5 ml [13]. Lower PSMs were reported for the intervention group 15% vs. 20% and even lower at the lesion 5% vs. 20% [13]. However, complications were higher in the 3D group, two patients vs. one [13].

Oderda et al. conducted a prospective pilot study to investigate the effectiveness of elastic fusion virtual models [10]. However, the effectiveness of the intervention is difficult to assess as this study lacks comparison to a control group. Overall, a short time was required to produce models—median 6 min and these did show to influence preoperative planning [10]. Six procedures were changed based on the model—displaying the usefulness of 3D models in preoperative planning [10]. The results indicate that the models could potentially increase surgical precision—no PSM% were reported (0%) [10].

### Meta-analysis

Only cohort studies were used for meta-analysis, whilst leaving the only RCT study separately, i.e. Shirk et al.’s study [11], which is concordant to the results of the meta-analysis for surgical positive margins (see prediction interval). Note that the prediction interval, which incorporates the between-study heterogeneity, crosses the line of no effect, suggesting that control groups could be superior to 3D models in a future study. Apart from surgical positive margins, there is no clear evidence of either groups favouring other postoperative outcomes.

### Limitations

The main limitations identified from this research come from the reported methodology, lack of printed models in comparison to AR and the lack of research comparing the two forms with each other. All studies have a limited methodology reported for model creation with common reporting being the slicing thickness on MRI and the software used. Only two studies selected used printed models however in comparison to the AR models they reported a decrease in complications whereas studies such as Porpiglia et al. reported higher complications in the 3D group [14]. To identify the prostate and tumour on medical images, an experienced radiologist was required to manually identify anatomy which can be time consuming. It is important to note the varying degrees of experience of each surgeon in the studies. Less-experienced surgeons may benefit the most from using the models compared to more senior experienced surgeons. We observe that this distinction is not accounted for in these studies. Another limitation is the lack of complications accounted for. Commonly blood loss, operative time, SHIM scores, PSM and continence were reported which allows for reliability of results to be determined; however, the background research highlighted other issues such as lymphocele/lymphorrhea, which were not investigated specifically. An improvement to be made based on this is to report on complications individually rather than solely report the number of them. Lastly, due to the lack of 3D-printed studies we were unable to compare 3D-printed vs. 3D-virtual models separately.

## Conclusion

The results of the literature gathered in this review present mixed findings. Overall, there is a promising amount of evidence that the use of 3D models does in fact lower complications and that there is a solid premise in this field to be investigated. General trends report reduced/lower operative times, continence was achieved at a good rate, and positive surgical margins do tend to be lower when a 3D model is used. In terms of qualitative data there was an apparent trend that the use of the surgical model was beneficial for surgeons—who had increased confidence in the procedure or had re planned the surgery. Virtual models tended to be more commonly used compared to printed models. Future studies are expected to investigate the effects of 3D models on surgical outcomes across different levels of surgeons’ experience, the number and justification of anatomical structures to include, and during what RARP surgical steps 3D models could be the most informative to surgeons.

## Supplementary Information

Below is the link to the electronic supplementary material.

Supplementary file1 (DOCX 36 KB)

## Data Availability

No data are shared with this manuscript.
