# Supplementary material for: Reduction of surgical complications via 3D models during robotic assisted radical prostatectomy: review of current evidence and meta-analysis
Source: J Robot Surg. 2024 Aug 6;18(1):304. doi: 10.1007/s11701-024-02041-2 (PMC11303509; doi:10.1007/s11701-024-02041-2)
Supplement: Supplementary file 1 — Supplementary file1 (DOCX 36 KB) [file 11701_2024_2041_MOESM1_ESM.docx]

**Reduction of Surgical Complications via 3D Models during Robotic-assisted Radical Prostatectomy: Review of Current Evidence and Meta-Analysis**

**Supplementary Material**

Kenaan Sarhan†^3^, Nawal Khan†^1,3^, Davide Prezzi^1,3^, Michela Antonelli^1^, Eoin Hyde^5^, Findlay Macaskill^3^, Christopher Bunton^2^, Nick Byrne^2^, Andres Diaz-Pinto^1,6^, Armando Stabile^4^, Alberto Briganti^4^, Giorgio Gandaglia^4^, Nicholas Raison^1,3^, Francesco Montorsi^4^, Sebastien Ourselin^1^, Prokar Dasgupta^1,3^, Alejandro Granados^1^

^1^ School of Biomedical Engineering & Imaging Sciences, King’s College London, London, UK

^2^ Medical Physics and Clinical Eng, Guy’s and St Thomas’ NHS Foundation Trust, London, UK

^3^Department of Urology, Guy’s Hospital, London, UK

^4^ Unit of Urology, San Raffaele Hospital, Milano, Italy

^5^ Innersight Labs, London, UK

^6^ NVIDIA Santa Clara, CA, US

† These authors contributed equally to this work

Corresponding author: Alejandro Granados

alejandro.granados@kcl.ac.uk

(https://orcid.org/0000-0002-2866-1324)

**Abstract**

*Motivation*: The use of 3-Dimensional (3D) technology has become increasingly popular across different surgical specialities to improve surgical outcomes. 3D technology has the potential to be applied to robotic assisted radical prostatectomy to visualise the patient’s prostate anatomy to be used as a preoperative and peri operative surgical guide. *Methodology*: This literature review aims to analyse all relevant pre-existing research on this topic. Following PRISMA guidelines, a search was carried out on PubMed, Medline, and Scopus. *Results*: A total of 7 studies were included in this literature review; 2 of which used printed 3D models and the remaining 5 using Virtual Augmented Reality (AR) 3D models. *Conclusion*: Results displayed variation with select studies presenting that the use of 3D models enhances surgical outcomes and reduces complications whilst others displayed conflicting evidence. *Significance*: The use of 3D modelling within surgery has potential to improve various areas. This includes the potential surgical outcomes, including complication rates, due to improved planning and education.

**Keywords**: Robot assisted radical prostatectomy, 3D printed models, 3D virtual models, Surgical complications

**Table 1. Three main components of our literature review with their corresponding topics. These topics were searched across PubMed, Medline, and Scopus to determine the most effective search term combination.**

| **Component** | **Topics** |
| --- | --- |
| **3D models** | 3D prostate models  3D printed prostate models 3D virtual prostate models |
| **Prostatectomy** | Radical prostatectomy Robotic prostatectomy  Robotic assisted radical prostatectomy |
| **Complications** | Prostatectomy complications  Robotic assisted radical prostatectomy complications |

**Table 2. Number of hits for all search topics (Table 1) across each of the selected databases.**

| **Search Term** | **Number of hits** | | |
| --- | --- | --- | --- |
|  | **PubMed** | **Medline** | **Scopus** |
| **3D prostate models** | 1,365 | 1,615 | 1,699 |
| **3D printed prostate models** | 69 | 42 | 55 |
| **3D virtual prostate models** | 65 | 66 | 92 |
| **Radical prostatectomy** | 26,567 | 26630 | 30,241 |
| **Robotic prostatectomy** | 5841 | 5092 | 6,405 |
| **Robotic assisted radical prostatectomy** | 3839 | 3225 | 3,920 |
| **Prostatectomy complications** | 12,788 | 10,449 | 12,597 |
| **Robotic assisted radical prostatectomy**  **complications** | 1,575 | 1,369 | 1,705 |
| **3D models and prostatectomy** | 148 | 29 | 179 |
| **3D models and radical prostatectomy** | 106 | 22 | 118 |
| **3D models and robotic assisted radical**  **prostatectomy** | 30 | 7 | 30 |
